# Regression of *ETV6-NTRK3* Infantile Glioblastoma After First-Line Treatment With Larotrectinib

**DOI:** 10.1200/PO.20.00017

**Published:** 2020-06-30

**Authors:** Musa Alharbi, Nahla Ali Mobark, Ali Abdullah O. Balbaid, Fatmah A. Alanazi, Wael abdel Rahman Aljabarat, Eman A. Bakhsh, Shakti H. Ramkissoon, Malak Abedalthagafi

**Affiliations:** ^1^Department of Paediatric Oncology, Comprehensive Cancer Centre, King Fahad Medical, Riyadh, Saudi Arabia; ^2^Radiation Oncology Department, Comprehensive Cancer Centre, King Fahad Medical City, Riyadh, Saudi Arabia; ^3^Department of Clinical Pharmacy, King Fahad Medical City, Riyadh, Saudi Arabia; ^4^Radiology Department, King Fahad Medical City, Riyadh, Saudi Arabia; ^5^Foundation Medicine, Morrisville, NC; ^6^Wake Forest Comprehensive Cancer Center and Department of Pathology, Wake Forest School of Medicine, Winston-Salem, NC; ^7^Genomics Research Department, Saudi Human Genome Project, King Fahad Medical City and King Abdulaziz City for Science and Technology, Riyadh, Saudi Arabia

This case study demonstrates that first-line treatment of a neurotrophic receptor tyrosine kinase (*NTRK*) fusion–positive infantile glioblastoma with larotrectinib, an NTRK inhibitor (NTRKi), was a safe and effective way to achieve tumor regression in our patient. An 18-month-old Saudi Arabian female presented with a history of right-sided weakness and partial seizures. Brain magnetic resonance imaging (MRI) revealed a large left frontal complex contrast-enhancing mass. Craniotomy for gross total resection (GTR) was performed, and histologic pathologic analysis revealed a diagnosis of glioblastoma. Postoperatively, the patient showed excellent recovery with no neurologic deficits. The tumor was then submitted for comprehensive genomic profiling. As a result of the expected poor survival, the patient’s family declined standard therapy, including chemotherapy and/or radiation therapy. Molecular analysis reported an *ETV6-NTRK3* fusion, which can be targeted by larotrectinib, an oral tyrosine kinase (TRK) inhibitor. At the 3-month postresection follow-up, MRI showed local tumor recurrence. Given the results of molecular testing, the family agreed to initiate oral larotrectinib as a less invasive therapy. Follow-up MRI was performed 8 weeks after larotrectinib treatment and showed significant tumor regression, indicating a positive response to treatment with no reported adverse effects. This patient case highlights the importance of genomic profiling for pediatric brain tumors to identify targetable alterations. It further demonstrates the potential for TRK inhibitors as a first-line therapy for malignant pediatric brain tumors harboring TRK fusions.

## PATIENT PRESENTATION

**FIG 1.**
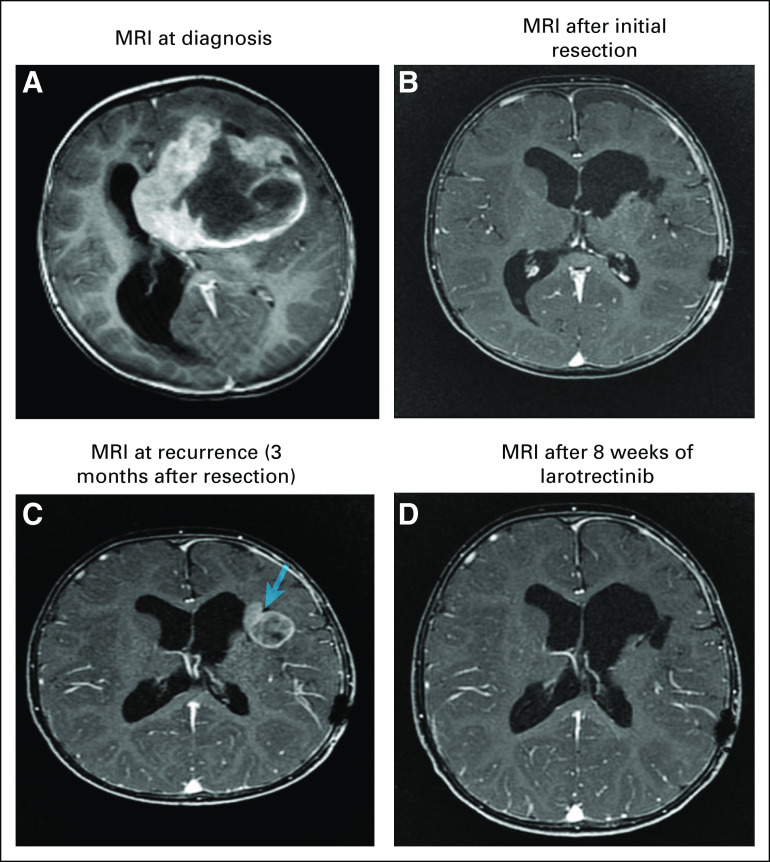
(A) At diagnosis, postgadolinium axial T1-weighted magnetic resonance imaging (MRI) demonstrated a heterogeneous complex mass in the left frontal lobe. (B) After total surgical resection, postgadolinium axial T1-weighted MRI demonstrated total resection of the left frontal mass. (C) At recurrence, postgadolinium axial T1-weighted MRI demonstrated newly appearing mass within the operative bed (arrow) showing intermediate diffusion signal with corresponding contrast enhancement indicating recurrent tumor. (D) Eight weeks after larotrectinib, follow-up postgadolinium axial T1-weighted MRI demonstrated disappearing left frontal mass indicating significant treatment response

In March 2019, an 18-month-old female child was admitted with right-sided weakness and partial seizures for 4 weeks. MRI showed a large, heterogeneous, complex left frontotemporal mass with internal cystic and necrotic changes with mass effect and a rightward midline shift with enhancing focus at the left posterior thalamic region (8 × 7 × 9 cm; Fig 1A). Whole-spine imaging was unremarkable, and the patient underwent craniotomy for GTR. The patient recovered well from surgery with no neurologic deficits. Postoperative MRI confirmed GTR but did note a small focus of nodular enhancement in the left insular cortex suggestive of residual tumor (Fig 1B).

**FIG 2.**
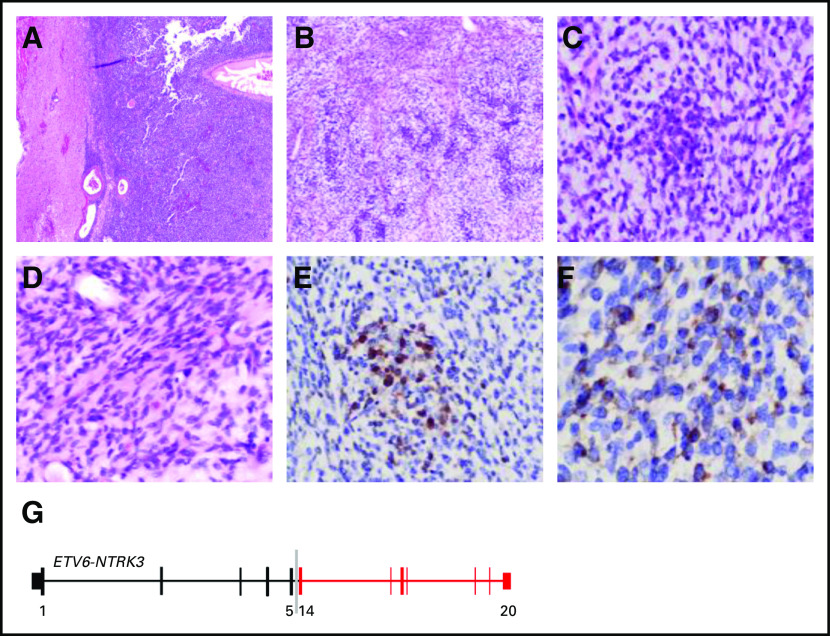
(A) Sheet of highly cellular monomorphic round primitive cells with relative demarcation from adjacent brain tissue (hematoxylin and eosin [HE]; original magnification, ×20). (B) Heterogeneous morphologic features of the neoplasm composed of focal areas of spindle cells arranged in small fascicles and primitive round cells in myxoid background with alternating hyper- and hypocellularity (HE; original magnification, ×200). (C) Higher magnification of the primitive cells in the myxoid background (HE; original magnification, ×200). (D) Higher magnification of the spindle cell area (HE; original magnification, ×20). (E) Few neoplastic cells show cytoplasmic and nuclear immunostaining for S100 (S100; original magnification, ×200). (F) Focal areas show neoplastic cells with cytoplasmic staining of synaptophysin (synaptophysin; original magnification, ×400). (G) Schematic of *ETV6-NTRK3* fusion detected in patient’s tumor.

Neuropathologic analysis of resected tissues revealed features consistent with a high-grade glioma best classified as glioblastoma (WHO grade IV). The tumor was composed of hypercellular sheets of primitive round to oval cells with fine open chromatin and no visible nucleoli (Fig 2A). The growth pattern consisted of intermixed small fascicles of spindle cells and haphazardly arranged primitive round cells in a myxoid background associated with delicate vasculature (Fig 2B). There were areas of necrosis and frequent mitosis. The neoplastic cells were diffusely immunopositive for vimentin; were focally positive for S100 and synaptophysin; showed retained nuclear staining for INI1; and were immunonegative for SMA, EMA, CD99, CD31, CD34, and NeuN (Figs 2C to 2F). Genomic profiling of tumor tissue revealed the presence of an *ETV6-NTRK3* fusion, which creates a novel chimeric oncoprotein that results in continuous activation of the NTRK3 kinase. The fusion encompassed exons 1 to 5 of *ETV6* and exons 14 to 20 of *NTRK3*, which retains the kinase domain of NTRK3 (Fig 2G).

As a result of the poor survival rates associated with glioblastoma, the patient’s family declined chemotherapy or radiation therapy despite extensive counseling on standard-of-care treatment. The patient remained stable with no adjuvant therapy until a 3-month postsurgery MRI showed local tumor recurrence and interval progression. The size of the recurrent lesion at the surgical bed was 2.3 × 2.8 × 2.7 cm and was associated with central necrosis (Fig 1C). Spinal cord and cauda equina imaging was unremarkable.

Given that larotrectinib (Vitrakvi; Bayer, Whippany, NJ), an oral TRK inhibitor, has been approved for treatment of advanced tumors in adult and pediatric patients with *NTRK* gene fusions,^1-7^ the patient was granted compassionate access to larotrectinib. In October 2019, the patient’s parents consented to oral larotrectinib (100 mg/m^2^ per day [50 mg/m^2^ given twice a day]) as a first-line therapy as a result of its convenience of administration, particularly in liquid formulations. Larotrectinib was well tolerated with no reported adverse effects and an excellent quality of life. MRI after 8 weeks of therapy showed marked tumor regression indicative of an excellent treatment response (Fig 1D). The patient has received continuous larotrectinib therapy since October 2019 and was scheduled for her 6-month follow-up MRI studies in April 2020; however, as a result of the global COVID-19 pandemic, the patient’s family has deferred further nonemergent hospital visits (including follow-up MRIs) until the self-isolation restrictions have been lifted. Therefore, the patient was advanced to a digital clinic visit, where she was noted to be clinically stable and neurologically intact without any evidence of adverse events or toxicities to date. We obtained consent from the patient’s guardian to publish the patient’s presentation and related images.

## METHODS

### Genomic Profiling

Next-generation sequencing was performed as previously described^8^ using the Oncomine Comprehensive Assay v3 system (Thermo Fisher Scientific, Waltham, MA). Copy number variants, single nucleotide variants, gene fusions, and indels were evaluated from 161 genes using multiplex DNA primers to prepare amplicon libraries. Assays were performed using the Ion S5 System and Ion 540 Chip (Thermo Fisher).

## DISCUSSION

Primary brain tumors are a leading cause of cancer-related morbidity and mortality in children.^9^ High-grade gliomas (HGGs) account for approximately 10% of pediatric brain tumors and are the second most common malignant CNS tumor after medulloblastoma. The most frequent tumors are anaplastic astrocytoma (WHO grade III) and glioblastoma (WHO grade IV).^10^ Glioblastoma occurs in both children and adults and is associated with a poor prognosis. Despite extensive studies in recent years, the clinical management of the tumors has remained largely unchanged, consisting of surgical resection, conventional chemotherapy, and radiotherapy.^8,9,11^ Young children treated with cranial irradiation experience devastating neurocognitive sequelae and tumor recurrence; however, there is growing evidence that younger patients (< 36 months old) demonstrate longer event-free and overall survival when treated with single-cycle induction chemotherapy consisting of vincristine, carboplatin, and temozolomide.^12^ However, progression-free survival rates remain poor for these patients, highlighting the need for new therapeutic options, including small molecules and/or immunotherapy alone or in combination with chemotherapy regimens.^13^ Although the etiology and genomic drivers of glioblastoma are diverse,^14-16^ a common finding in pediatric HGG, especially infantile HGGs, is the presence of fusions involving *NTRK*, *ALK*, and *ROS1*, among others. TRK fusion proteins are oncogenic drivers that have been reported in a wide range of adult and pediatric tumors that occur at high frequencies (≥ 90%) in rare cancer types.^17,18^ Gene fusions involving the kinase domain of each of the 3 neurotrophin receptors (NTRK1, NTRK2, and NTRK3) for different N-terminal fusion partners, were identified in 4% of diffuse intrinsic pontine gliomas and 10% of non–brain stem (NBS) HGGs. Notably, in one study, 4 (40%) of 10 NBS-HGGs in children younger than 3 years old harbored an *NTRK* fusion gene. The high frequency of *NTRK* fusions in NBS-HGGs from children age ≤ 3 years and the paucity of additional mutations in these tumors strongly suggest that the fusion genes are potent oncogenic drivers in early postnatal brain tumor development.^19^

*NTRK* fusions have been identified at low frequencies in low-grade pediatric astrocytomas and adult glioblastomas.^20,21^ TRK ligands (commonly nerve growth factor for TRKA, brain-derived growth factor or neurotrophin 4 for TRKB, and neurotrophin 3 for TRKC) bind with high affinity to the extracellular domain of the TRK receptor.^22,23^ This leads to receptor activation and the induction of signal transduction pathways involved in proliferation, differentiation, and survival (eg, MAPK, PI3K, and PKC pathways) in both normal and neoplastic cells.

The activity of the first-generation TRK-selective inhibitor larotrectinib in pediatric patients with tumors harboring NTRK fusions has been explored in clinical trials, including a phase I/II trial in pediatric patients (SCOUT; ClinicalTrials.gov identifier: NCT02637687) and a phase II trial involving adults and adolescents (NAVIGATE; ClinicalTrials.gov identifier: NCT02576431). Larotrectinib is a potent and selective inhibitor of all 3 TRKs, producing potent and well-tolerated responses in adult and pediatric patients with NTRK-rearranged tumors, with sustained tumor regression in > 90% of infants, children, and adolescents with TRK fusions at doses of 100 mg/m^2^ twice a day (maximum, 100 mg per dose).^1-7^ The most common adverse events include mild elevations of liver enzyme levels, cytopenias, and vomiting. The high solubility of larotrectinib permit its use in liquid formulations in young patients unable to swallow capsules.^5,24^ Increasingly, case reports in the literature demonstrate tolerance and clinical response to NTRKi in pediatric patients with HGG, even after chemotherapy and radiation treatment.^25^ These patient cases highlight the need for clinical trials that assess the sustained durability of response to NTRKi and whether these targeted therapies should be used as monotherapy agents or in combination with chemotherapy regimens.

Our patient case demonstrates that TRK inhibitors can be integrated as a first-line therapy for pediatric HGGs harboring TRK fusions. This approach spares the developing CNS from the secondary effects of combined chemotherapy and radiotherapy, which have uncertain efficacy, and many patients show incurable progression after conventional therapeutic modalities, with a dismal outcome. We also highlight the need for the integration of genomic profiling in the routine histopathologic analyses of pediatric patients with malignant primary intracranial tumors to detect any genetic mutations that can be targeted with available therapies. This can avoid the morbidity associated with nonprecision conventional therapies.
